# Accuracy of ChatGPT in answering cardiology board-style questions

**DOI:** 10.3352/jeehp.2025.22.9

**Published:** 2025-02-27

**Authors:** Albert Andrew

**Affiliations:** Department of Medicine, School of Medicine, The University of Auckland, Auckland, New Zealand; The Catholic University of Korea, Korea

Chat Generative Pre-trained Transformer (ChatGPT), a free generative artificial intelligence (AI) chatbot released by OpenAI, has sparked discussions about its potential in various industries, including medicine. In cardiology, it showed promise when tested on 25 cardiovascular questions based on clinical experience and guidelines [1]. It provided 21 appropriate responses on topics such as risk counseling, test interpretation, and medication details, as evaluated by preventive cardiologists [1]. A notable area of research has been evaluating ChatGPT’s accuracy in answering board-style questions for various specialist certification examinations. This mini-review with a meta-analysis examined ChatGPT’s performance on cardiology board-style questions in text and image formats across multiple versions, offering a clearer assessment of its impact on cardiology medical education.

A search for all published papers that reported ChatGPT’s performance on knowledge-based cardiology questions was conducted in PubMed/MEDLINE and EMBASE. The literature search was performed in December 2024 using the following search query: (“ChatGPT” OR “GPT-4” OR “GPT-3.5”) AND (“cardiology”) AND (“board” OR “certification” OR “specialty”). Studies were included in the analysis if they met all of the following criteria: (1) the article was written in English; (2) the study assessed ChatGPT’s accuracy on questions that were set at a level or retrieved from an appropriate resource representing board-style (specialist) cardiology certification examination questions; (3) the questions inputted into ChatGPT were either text-based, image-based, or a combination of both; and (4) the study provided data on the number of questions inputted into ChatGPT and the number (or percentage) of correct responses reported separately for each question format. Studies were excluded if they failed to meet any of the aforementioned inclusion criteria or did not disclose original data, such as review papers or descriptive replies/correspondence to previously published articles. Key study characteristic data from included studies were extracted and entered into a predefined data abstraction template. Statistical analysis pooled the reported accuracy from each study to calculate an overall pooled accuracy with a 95% confidence interval (CI), subgrouped by model version, using a random-effects model. The meta-analysis software used was STATA ver. 18.0 (Stata Corp.). P-values <0.05 were considered statistically significant. Heterogeneity was assessed using the I^2^ statistic.

Our initial search identified a total of 36 studies (25 from PubMed, 11 from EMBASE). After removing duplicates (n=9), all of the remaining 27 studies underwent full text screening. Applying the inclusion and exclusion criteria, 7 studies were ultimately included in the analysis. A summary of the key characteristics of each study is depicted in Table 1 [2-8]. The meta-analysis was divided into 2 parts: one analyzing the accuracy of text-based questions and the other for image-based questions. Accuracy was calculated using data from Dataset 1 and Dataset 2, with results further categorized by the ChatGPT version used in each study (Table 1).

For multiple-choice text-based questions (Fig. 1), data from 6 of the 7 studies were analyzed. The results showed that ChatGPT, across all versions, achieved an overall pooled accuracy of 58.64% (95% CI, 52.01%–65.13%; I^2^=84.41%, P=0.00). Among the versions, ChatGPT-3.5 Plus had the lowest performance, with an accuracy of 43.84% (95% CI, 36.28%–51.55%). In contrast, ChatGPT-4omini demonstrated the highest accuracy, achieving 66.70% (95% CI, 58.57%–73.95%). However, this result is based on a single study, limiting its representativeness in the subgroup analysis.

For multiple-choice image-based questions (Fig. 2), data from 3 of the 7 studies were included. The pooled accuracy for ChatGPT across all versions was 43.10% (95% CI, 35.74%–50.59%; I^2^=0%, P=0.41). ChatGPT-4.0 Plus performed the worst in this category, with an accuracy of 41.00% (95% CI, 31.87%–50.80%). In contrast, ChatGPT-4.0 achieved the highest accuracy for image-based questions, scoring 48.49% (95% CI, 30.50%–66.65%).

In our paper, ChatGPT’s performance was categorized into 2 areas: multiple-choice text-based and image-based questions. However, not all studies provided sufficient data for both categories. For example, Alexandrou et al. [8] included a breakdown of performance data (number of questions inputted and number of correct outputs) for image and video-based questions, but lacked similar details for text-based questions, preventing a complete assessment of ChatGPT’s performance in that area. This limitation may reduce the overall comprehensiveness and accuracy of our analysis.

The pooled accuracy of ChatGPT across all versions in this meta-analysis was 58.65% for text-based multiple-choice questions and 43.10% for image-based multiple-choice questions. Interestingly, there was a noticeable difference in performance for text-based questions between ChatGPT-3.5 and GPT-3.5 Plus, with accuracy of 56.42% and 43.84%, respectively, despite both versions being built on the same underlying training model (or architecture) [8]. While it is challenging to define a universal passing mark due to the variation in cardiology topics and difficulty levels across different examination jurisdictions, some studies have suggested that the minimum passing mark typically ranges from 60% to 73% [4,8]. Our meta-analysis suggests that ChatGPT, across all versions, is unlikely to achieve a passing score on cardiology board-style certification examinations for both text and image-based multiple-choice questions.

When ChatGPT’s performance on cardiology board-style certification questions was compared to that of successful human test-takers who had satisfactorily passed when faced with such questions, its accuracy was consistently inferior [3,6,8], with a difference in accuracy ranging from 5.5% to 12% [6,8]. This suggests that ChatGPT is not yet capable of performing at the level of human cardiologists on certification examinations and thus may lack the necessary clinical knowledge to make decisions as effectively as experienced cardiology clinicians.

Milutinovic et al. [3] also compared ChatGPT’s performance on board-style cardiology questions to that of trainee cardiologists, and non-cardiology-trained physicians. They found that, although ChatGPT may not have the same depth of knowledge as experienced cardiologists, it outperformed both trainee cardiologists and non-cardiology-trained physicians [3]. This finding is significant, as it suggests that ChatGPT, particularly the 4.0 version, could still be a valuable supplementary resource for medical students and cardiology trainees. It can help structure and apply cardiology knowledge in a systematic manner, assisting learners in understanding fundamental cardiology concepts by generating structured study aids, such as content maps for specific cardiology topics. This feature can be particularly beneficial for students and test takers, helping them organize complex information in a clear and systematic manner. Additionally, ChatGPT can support the creation of case-based learning materials and review articles, both of which are essential for continuous professional development [9]. Furthermore, examiners could use ChatGPT to generate clinical vignettes (or scenarios) for examination questions, making the process more cost-effective and less time-consuming compared to traditional methods. However, because ChatGPT lacks access to the latest clinical guidelines, it has a limited ability to generate accurate, up-to-date medical questions [10]. Manual adjustments may be needed to ensure the reliability and relevance of the generated scenarios and questions. Future studies should assess the completeness of ChatGPT’s responses to real-world cardiology cases and their relevance across different training systems and guidelines.

A key limitation of this review is the restricted search strategy, as only 2 databases were used. This may have led to the exclusion of relevant studies. Expanding the search to include additional databases and using more comprehensive keywords or search queries could have captured a wider range of literature, thereby strengthening the robustness of this analysis. In some studies that reported both text-based and image-based accuracy outcomes, there was often insufficient data to calculate one of the 2 outcomes. As a result, only one of the 2 results was included in our analysis for those studies. Moreover, significant heterogeneity was observed in ChatGPT’s accuracy on text-based questions (I^2^=84.41%). This indicates that effect sizes vary, reflecting inconsistencies in ChatGPT’s accuracy. Heterogeneity arises from factors such as question difficulty and language, as well as the phrasing of the input prompt, all of which influence how the model interprets and responds to queries. In contrast, no heterogeneity was observed in image-based questions, likely because they rely on more standardized visual features rather than variable linguistic inputs.

Nevertheless, beyond ChatGPT’s accuracy in answering cardiology board-style questions, the future of AI in medical education and assessment looks promising. Its impact extends widely across all areas of continuing medical education, influencing virtually every medical discipline [11]. A scoping review identified various AI applications in medical education, from basic uses like personalized learning and feedback platforms to more advanced innovations such as virtual trainers and simulators for assessment, as an alternative to human observation and feedback [12]. Interestingly, while physicians and medical students generally have a positive attitude toward AI in continuing education, relatively few medical students and clinicians have direct experience with its use and associated technologies [11]. Therefore, to address this, future research should focus on developing universal protocols that are capable of rigorously validating various AI tools available in the medical educational sphere, ensuring their effectiveness and usability for current and future medical professionals.

## Figures and Tables

**Fig. 1.**
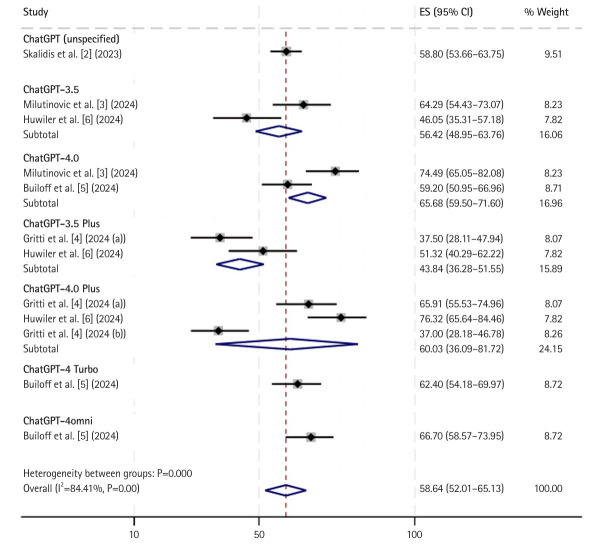
Accuracy of ChatGPT in answering multiple choice text-based cardiology board-style (specialty) questions sub grouped by different model versions. ES, estimate; CI, confidence interval.

**Fig. 2.**
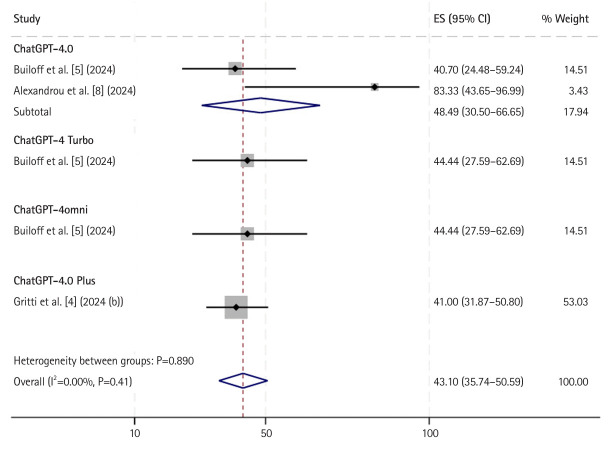
Accuracy of ChatGPT in answering multiple choice image-based cardiology board-style (specialty) questions sub grouped by different model versions. ES, estimate; CI, confidence interval.

**Table 1.** Summary of study characteristics, ChatGPT versions, question set descriptions, and overall accuracy

First author surname	Year of study	Version(s) of ChatGPT assessed	Description of question set	Overall reported accuracy
Skalidis et al. [2]	2023	ChatGPT (unspecified)	The dataset includes a total of 362 text-based multiple-choice questions sourced from various materials. This includes 68 questions derived from European Society of Cardiology sample resources, 144 questions from the 2022 edition of StudyPRN test questions, and another 150 questions from Braunwald’s Heart Disease Review and Assessment textbook.	Text-based accuracy: ChatGPT-3.5=58.8% (n=312/362)
Milutinovic et al. [3]	2024	ChatGPT-3.5 and ChatGPT-4.0	98 Text-based multiple-choice questions were chosen from a selected Cardiovascular Medicine chapter within the Medical Knowledge Self-Assessment Program (MKSAP-19).	Text-based accuracy: ChatGPT-3.5=64.3% (n=63/98); ChatGPT-4.0=74.5% (n=73/98)
Gritti et al. [4]	2024 (a)	ChatGPT-3.5 Plus and ChatGPT-4.0 Plus	88 Text-based multiple-choice questions from the Paediatric Cardiology Board Review textbook	Text-based accuracy: ChatGPT-3.5 Plus=37.5% (n=33/88); ChatGPT-4.0 Plus=65.9% (n=58/88)
Builoff et al. [5]	2024	ChatGPT-4, ChatGPT-4 Turbo, and ChatGPT-4omni (GPT-4o)	168 Multiple-choice questions (141 text-only and 27 image-based) from the 2023 American Society of Nuclear Cardiology Board Preparation Exam	Text-based accuracy: ChatGPT-4.0=59.2%; ChatGPT-4 Turbo=62.4%; ChatGPT-4omni (GPT-4o)=66.7%
				Image based accuracy: ChatGPT-4.0=40.7%; ChatGPT-4 Turbo=44.4%; ChatGPT-4omni (GPT-4o)=44.4%
Huwiler et al. [6]	2024	ChatGPT-3.5, ChatGPT-3.5 Plus, and ChatGPT-4.0 Plus	The dataset features 88 multiple-choice questions from the Switzerland Cardiological Board Exam. Of these, 76 were text-based questions and 12 were image-based questions. These questions cover various cardiology topics and subfields and are based on the 10th Edition of Braunwald’s Heart Disease Review and Assessment.	Text-based accuracy: ChatGPT-3.5=46.05% (n=35/76); ChatGPT-3.5 Plus=51.30% (n=39/76); ChatGPT-4.0 Plus=76.3% (n=58/76)
				Image-based accuracy: unable to be determined
Gritti et al. [7]	2024 (b)	ChatGPT-4.0 Plus	100 Multiple-choice questions with and without accompanying images from the Paediatric Cardiology Board Review textbook	Image based accuracy: ChatGPT-4.0 Plus=41.0% (n=41/100)
				Text-based accuracy: ChatGPT-4.0 Plus=37.0% (n=37/100)
Alexandrou et al. [8]	2024	ChatGPT-4.0	60 Multiple-choice questions were included from the CATHSAP platform. This resource simulates the American College of Cardiology/Society for Cardiovascular Angiography and Interventions international cardiology certification examination, providing a robust preparation tool for candidates	Text-based accuracy: unable to be determined
				Image based accuracy: ChaGPT-4.0=83.3% (n=5/6)

## Footnotes

**Data availability**
Data files are available from Harvard Dataverse: https://doi.org/10.7910/DVN/MYQKNY Dataset 1. ChatGPT cardiology board exam accuracy data for text-based questions.

Dataset 2. ChatGPT cardiology board exam accuracy data for image-based questions.
